# Application of Laser Vibrometry to Assess Defects in Ship Hull’s Welded Joints’ Technical Condition

**DOI:** 10.3390/s21030895

**Published:** 2021-01-29

**Authors:** Adam Szeleziński, Adam Muc, Lech Murawski, Marcin Kluczyk, Tomasz Muchowski

**Affiliations:** 1 Department of Maritime Engineering, Gdynia Maritime University, 81-225 Gdynia, Poland; l.murawski@wm.umg.edu.pl; 2 Department of Electrical Engineering, Gdynia Maritime University, 81-225 Gdynia, Poland; a.muc@we.umg.edu.pl (A.M.); 44349@student.umg.edu.pl (T.M.); 3 Mechanical-Electrical Department, Polish Naval Academy of the Heroes of Westerplatte, 81-103 Gdynia, Poland; m.kluczyk@amw.gdynia.pl

**Keywords:** structural health monitoring, non-destructive testing, optical vibrometry

## Abstract

The paper presents the measurement process and test results for six thin-walled plates with different dynamic characteristics caused by different defects of welded joints. The tests were carried out using non-destructive testing (NDT). The authors made an attempt to determine the validity of the use and degree of effectiveness of the tests based on laser vibrometry in detecting defects in welded joints. The tests of welded plates were carried out using displacement laser sensors and piezoelectric accelerometers, while the source of vibration extortion was a modal hammer. In the adopted measurement methodology, the application of accelerometers was to obtain the reference data, which allowed for comparison with the measurement data obtained from the laser vibrometer. The analysis of the obtained measurement data, in the fields of time and frequency, made it possible to verify the correctness of the data obtained by means of laser vibrometry and to determine the requirements which are necessary for the correct performance of NDT tests and in the future structural health monitoring (SHM) system of welded joints with the use of a laser vibrometer. The mathematical model developed in the MSC software Pastran-Nastran was also used in the work. The model was developed for the purpose of mutual verification of the measurement and calculation tests. At the present stage of work, it can be stated that the results obtained by laser vibrometry methods should be treated as a supplement to the research conducted with traditional piezoelectric accelerometers. In certain situations, they can be used as an alternative to accelerometers, due to the fact that laser sensors do not require direct contact with the examined object. Where the object under test may be in a strong electromagnetic field, optical sensors are better suited than contact sensors.

## 1. Introduction

The main causes of defects in welded joints are deviations from the correct welding technology, improper selection and mispreparation of basic and additional materials for welding [1,2,3,4]. Other important factors influencing the quality of welds include non-technological structural solutions of joints, non-observance of the welding technology, insufficient qualifications and irresponsibility of welders and malfunctioning of welding equipment [5,6]. Knowledge of the reasons for the occurrence of inconsistency allows for easier and more precise interpretation of the quality of the tested joints, assessment of the impact of such inconsistency on the operational properties of joints and taking action to eliminate defects in the process of manufacturing welded structures [6,7]. Welding defects due to the mechanism of their occurrence are divided into technological defects, which are directly related to the manufacturing method and arise during an incorrectly performed technological operation (e.g., lack of re-melting, sticking) and operational defects caused by the working conditions of the element (e.g., fatigue cracks). Regardless of the reasons for the defect in the weld, failure to detect it in the production process or release for use can lead to serious consequences [2]. Both the safety of the structure and human life may be at risk.

The purpose of using non-destructive testing (NTD) is to determine the type, size and location of inconsistencies (defects in the welded joint) in order to determine their acceptance or the necessity of removing them from the tested item [1,8,9]. Inspection activities to ensure the quality of welded joints are carried out both during the production process and during operation (structure monitoring). Failure to carry out in-service non-destructive testing of technical facilities such as aircraft, ships, lifting equipment or bridges, or to carry it out in accordance with the test procedures, may lead to serious catastrophes and accidents.

NDT testing techniques are well researched and give results with usually sufficient reliability [10,11,12,13,14]. However, they have a fundamental disadvantage—they are carried out periodically. In the periods between the tests, the reliability of the structure is unknown. Especially in case of critical events such as a strong storm or collision, it is important to know the possibility of further exploitation of the object. This information must include the degree of potential disaster risk together with the parameters of exploitation with particular emphasis on the time of safe use of the object [13,14,15,16]. To this end, research on new techniques known as structural health monitoring (SHM) is being developed [1,5,17,18,19,20]. SHM systems use a number of advanced sensors, such as fiber Bragg grating (FBG) sensors [21,22,23,24]. There are also works connected with the application of laser vibrometers in SHM application [8,25,26,27,28,29,30,31], but authors have not found any information about the use of such sensors onboard ships.

A wide range of different measurement techniques and methods are used in marine NDT and SHM techniques. What is more, proper optimization and processing of the obtained measurement signals are extremely important [32]. It is also necessary to “teach” the automatic fault recognition system, both in NDT and SHM examinations [33]. Especially ship structures require precise recognition of their characteristics [34] due to untypical marine constructions [35] and the strong interference of measurement signals by the sea environment. Numerous researchers have conducted trials with various measurement methods, ranging from: electromagnetic sensors [36], ultrasonic-guided waves [37], vibrothermography [38] and fibreoptic techniques (mostly FBG sensors) [39,40].

The aim of this article is to check whether SHM systems based on accelerometers—relatively simple and cheap sensors—can be useful. The authors are interested in the development of SHM systems in shipbuilding [28,41].

Typical dynamic characteristics can be determined for each object [21,42,43]. The distribution of resonance frequencies for the tested and undamaged elements should be constant during the operation of the object. The frequency and amplitude of vibrations in resonance are directly influenced by the mechanical characteristics of the tested object: mass, stiffness, damping factor [44]. A change in one or more of the above elements results in a change in the element’s response to extortion. The difference between the resonance characteristics of the tested object and the resonance characteristics of the reference object may inform us about the occurrence of a fault in the tested object.

The authors see the need to implement a fast and reliable method of assessing the correctness of welded joints in a ship’s hull elements, which can be carried out automatically during the ship’s operation [9,45]. The authors attempted to adapt laser vibrometry for this purpose.

## 2. Materials and Methods

Laser vibrometry allows carrying out measurements using a laser beam [26,27,31,46]. It enables measurements of vibrations in a non-contact and non-invasive way. This means that the tests do not affect the examined object in any way. The main principle of the laser vibrometer is that the frequency of vibrations of the tested surface can be measured by analyzing the reflected laser beam. The physical phenomena used in the measurements are interference and the Doppler effect. Laser vibrometry allows detection of high-frequency vibrations with high accuracy. As measurements are made optically, laser vibrometry is resistant to external interference in the form of a strong electromagnetic field. The main limitation in the application of vibration parameters measurements with the use of laser devices is the influence of significant air pollution on the accuracy of the measurements (in extreme cases, it is not possible to perform measurements at all). The second important limitation is the need to place the vibrometer on a stable and non-vibrating surface to avoid measurement errors.

**Figure 1:**
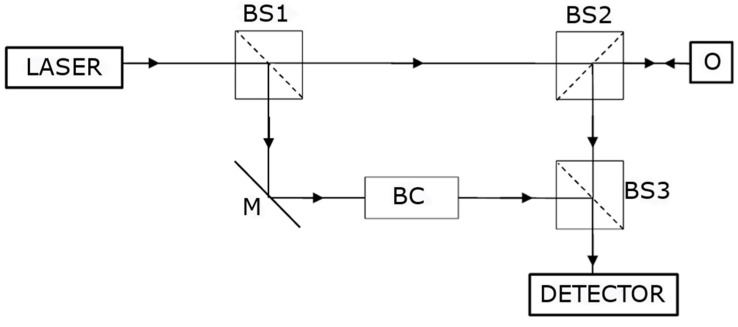
Diagram of the Doppler laser vibrometer measurement system, where M is a mirror, BS is a beam splitter, BC is a Bragg cell and O is the object under investigation.

Vibration tests with the use of laser vibrometry are often carried out by using the Doppler effect [8]. The laser beam focused on the tested surface will be shifted if the tested surface moves at a certain speed. By knowing the distribution of the vibration velocity, the vibration frequency can be calculated. The use of interferometry (e.g., Michelson interferometer) allows detecting the Doppler frequency shift, which correlates with the speed and frequency of vibrations [47]. In practice, in the beam splitter, a laser beam reflected from the examined object is applied to the control beam. This creates a changing pattern of interference on the detector, which will vary depending on the vibration of the examined object. The data of vibration parameters are recovered using the interference pattern by measuring the distance between the interference strips or by measuring the light intensity. Figure 1 shows a diagram of the Doppler laser vibrometer measurement system.

The operation of the measuring system shown in Figure 1 is as follows. The monochromatic light emitted by the laser is separated into two parts by the first beam splitter. The test beam hits the object after passing through the second beam splitter. The control beam passes through the Bragg cell. The object reflects the test beam and it passes through the third beam splitter, where it is superimposed on the control beam and then it goes to the detector, where an interference pattern is created in accordance with the movement of the object. The total intensity falling on the detector is expressed by Equation (1):

(1) $$I=\;I_{1}+\;I_{2}+2\sqrt{I_{1}I_{2}}\cos\left[\frac{2\pi \left(r_{1}-r_{2}\right)}{\lambda }\right]$$

where *I*_1_ is the intensity of the control beam, *I*_2_ is the intensity of the test beam, *r*_1_ is the optical length of the control beam path, *r*_2_ is the optical length of the test beam path and *λ* is the wavelength of the laser beam. The result of the above equation is interference, which describes the change in intensity created by the difference in the distance of the test and the control beam path. The task of the Bragg cell is to modify the interference pattern depending on the direction of the object’s movement (without the Bragg cell, the pattern would be identical for both directions of movement), whereas if the information about the direction of movement is insignificant in the context of the conducted study, the use of the Bragg cell is optional.

**Figure 2:**
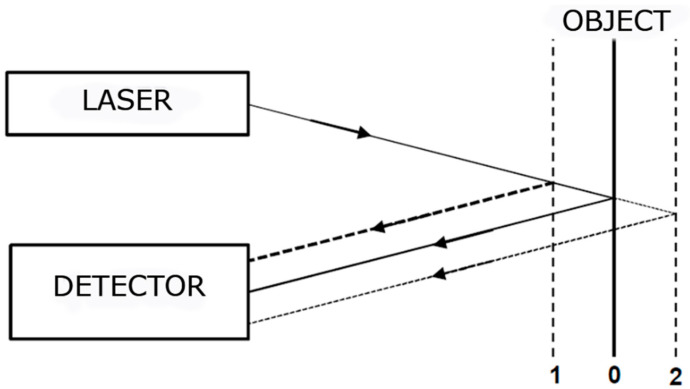
Diagram principle of the operation of a passive laser vibrometer.

Despite the much greater sensitivity and frequency range of detected vibrations, Doppler laser vibrometry is used much less frequently in SHM and NDT than passive laser vibrometry. The reason is the greater complexity of the system construction, which means higher costs of the system itself, but also of the measurements. Passive laser vibrometry has a much simpler operating principle than interferometer laser vibrometry. The measuring device consists of two parts—a laser and a detector. The laser beam is directed at the vibrating object and the reflection goes to the detector. A change in intensity in the reflected beam from a vibrating object is detected. During vibration, the object changes its position. When the object is further away from the measuring device (position 2), the distance traveled by the laser beam is greater, which means that the intensity of the reflected beam is lower. The situation is reversed if the object is in a closer position (position 1) to the measuring device. The principle of operation is illustrated in Figure 2.

The change in beam intensity is due to the effect of the law of inverted squares, which explains that the intensity of a light source is inversely proportional to the distance traveled. The law of inverted squares might be represented by Equation (2):

(2) $$I=\frac{1}{d^{2}}$$

where *I* is the intensity of light and *d* is the distance traveled.

In order to carry out the measurement with a passive laser vibrometer correctly, it is necessary to set it properly in relation to the examined object. The distance and angle between the measuring device and the object is important. An incorrect distance or angle makes it impossible to correctly measure the reflected laser beam on the detector’s surface, which may lead to a misrepresentation if the beam only partially reflects on the detector’s surface (only in certain positions of the object during its vibration). For this reason, the specification of devices for measuring vibrations by passive laser vibrometry often includes the information with details of the correct setting of the measuring devices.

In order to determine the usefulness of the proposed measurement methods in the analysis of the technical condition of plates’ welded joints of thin-walled structures, 6 plates of the same dimensions, i.e., 500 × 500 × 4 mm, made of the same material, i.e., DH36 steel, were tested. The welds were made by the same welder and the plates were plasma cut. Welding was carried out with TIG method, where the edges were chamfered before welding and welding was carried out in position 1 G with 1 bead joint. In most cases, joints with welds unsuitable for further use were selected. The joints were evaluated using the radiographic method in an external accredited laboratory according to standard 17636-1 and EN 5817. Due to the fact that, in international standards, there are just criteria of acceptance or rejection of welded joints to describe the degree of damage, two RTG/NDT inspectors were asked to determine it. Therefore, the percentage values of damage given below are subjective but based on many years of RTG/NDT inspectors’ work experience. The following types of damage to the welds were detected (the estimation of the strength of the welded joint in relation to the parent material is given in brackets):

- P1 plate (100%)—plate without welded joints (reference);
- P2 plate (70%)—no full melting;
- P3 plate (95%)—a few spherical blisters; according to the guidelines of classification societies, the combination is considered good;
- P4 plate (85%)—no full melting;
- P5 plate (80%)—no full melting;
- P6 plate (55%)—no full melting, edge gluing, longitudinal bladder.

**Figure 3:**
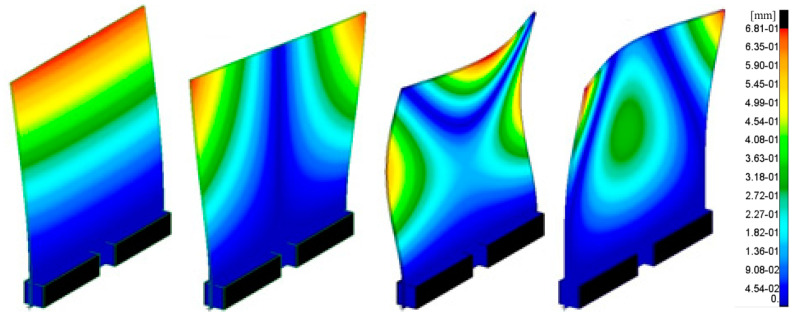
From the left: first, second, third and fourth forms of natural vibration of the analyzed plate.

Before starting the measurement tests, a mathematical model of a plate without a weld was made in order to determine its basic natural frequencies. This made it possible to precisely define the conditions and experimental research program. The MSC software Patran-Nastran environment was used for this purpose. The plate and the mounting were modeled using 3D solid hexagonal elements with eight nodes per element (Hex 8). A model with 407586 finite elements and 1572795 degrees of freedom was obtained. The size of the model was the same as the real object, i.e., 500 × 500 × 4. The modal analysis allowed determining the forms of the system’s own vibrations. From the research point of view, only the first four forms shown in Figure 3 are relevant. The next forms of natural vibration have values above 180 Hz. These forms are hardly enforceable in the real situations; additionally, they have small amplitudes which are usually unable to damage the object.

Even higher forms of natural vibration, starting with the sixth one, are difficult to force with frequencies exceeding 300 Hz. Difficulties with extortion of higher forms result from more nodal vibration lines. Therefore, the number of local amplitudes of vibrations is higher. In order to force such complex forms of vibration, it would be necessary to apply at least a few forces and moments remaining in the relevant phase relationships. In further analyses, only the first two forms of natural vibration will be considered. This decision was supported by preliminary analyses of the measurement tests. These were carried out using a laser vibrometer. The analysis of the measurements showed that the first two forms of the object’s own vibration are clearly observable.

The adopted measurement methodology assumed simultaneous measurement of vibration parameters of the plates, which were enforced by a modal hammer. The assumption is to use accelerometers only in order to verify the correctness of the values obtained during the measurements with the use of a laser vibrometer. Such an approach is to serve the subsequent easy examination of repetitive elements which could be tested in the technological sequence without the necessity of installing accelerometers [29]. The laser vibrometer allowing non-contact measurement is perfectly suited for such an application [48].

**Figure 4:**
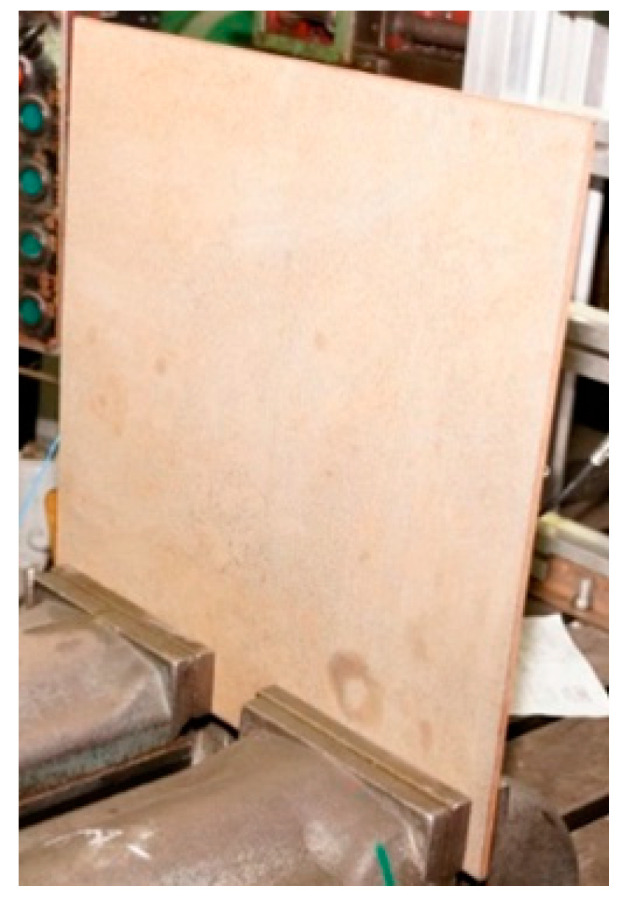
P1 plate (without welded joints) mounted on a test bench.

Particular attention was paid to the way in which the plates were mounted in order to ensure the greatest possible repeatability of the tests an example of a plate mounted on a test bench is shown in Figure 4.

**Figure 5:**
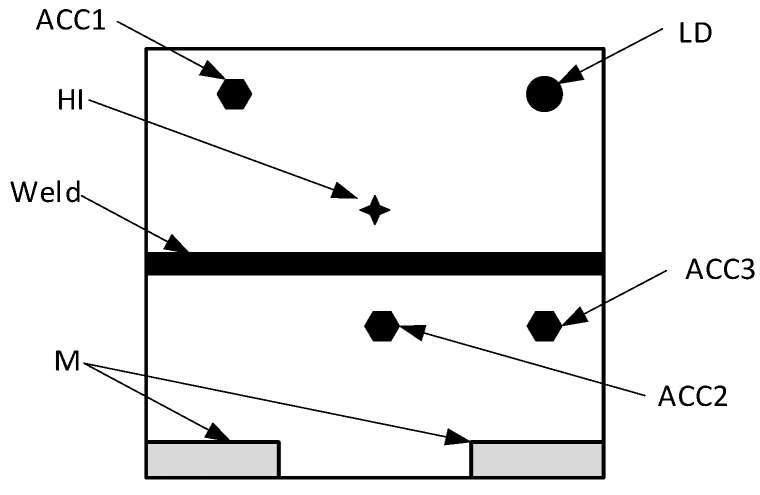
The arrangement of sensors during the second series of tests. ACC1–3—accelerometers, HI—place of impact with a modal hammer, M—mountings, LD—vibrometer laser spot.

The measurements were carried out according to the scheme shown in Figure 5. Enforcement with a modal hammer was applied in the marked place. The hammer was operated manually, so it can be concluded that the place of application of the forced impact pulse was not fixed both in terms of location and the value of the extortion. This simulates the actual working conditions of the planned structure monitoring system. The recording of the signals was repeated ten times for each plate. The aim was to check both the repeatability of the measurement results and the possibility of testing their errors and spreads.

**Figure 6:**
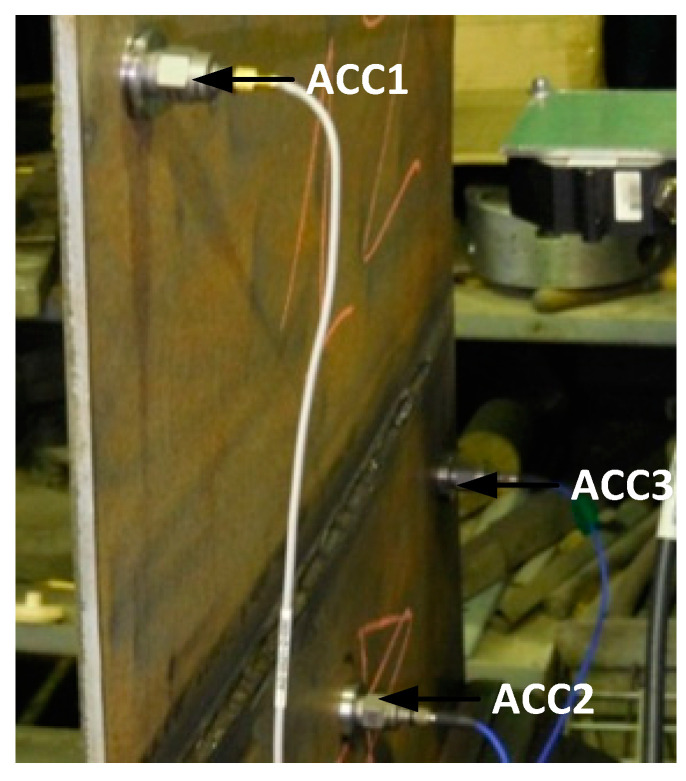
Picture of the tested plate on a measuring station with the accelerometers installed.

The signals from the modal hammer and accelerometers were recorded using a B&K measuring system consisting of a B&K 3050-A-60 cassette, 4514 B accelerometers and a B&K 2270 type modal hammer. The apparatus was configured in such a way that it was possible to automatically detect sensor overloads and double impacts of the modal hammer. Accelerometers mounted on one of the tested plates are shown in Figure 6.

**Figure 7:**
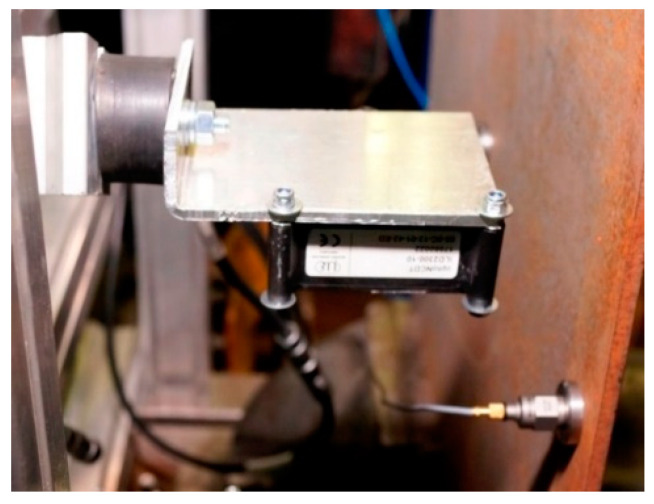
Laser displacement sensor on the test bench.

Simultaneously to the measurements of amplitudes of vibration accelerations, the measurements of amplitudes of plate displacements after the impact with a modal hammer were performed. The registration of plate position changes was carried out with the use of an optoNCDT triangulation laser sensor type ILD2300-10. In order to eliminate possible vibrations of the measuring element that could negatively affect the obtained results, the laser sensor was mounted on a special bracket, using a metal-rubber shock absorber (Figure 7). The sensor was located 35 mm from the tested plates.

**Figure 8:**
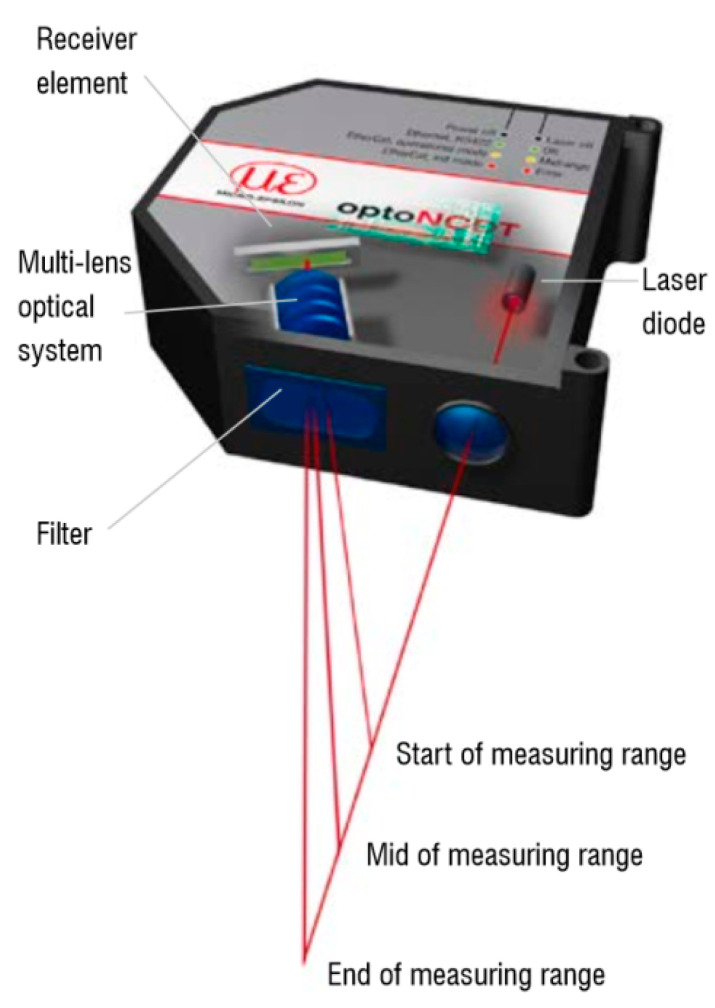
Measurement principle using a laser displacement sensor [49].

The laser sensor used for the non-contact displacement measurement works on the principle of triangulation which was already described (Figure 2 and Figure 8). This direct method of the displacement measurement does not cause an integration error and is therefore preferable to the displacement determination based on accelerometric acceleration measurements.

The applied laser sensor is characterized by high resistance to external interferences, as well as the built-in electronics which determine the position of the object with a sampling frequency up to 49.02 kHz. At the output of the sensor, a discrete voltage signal is obtained. The basic data of the sensor ILD2300-10 are shown in Table 1.

There was the assumption that the distance of the source of light in the laser vibrometer will not exceed the measuring range of the sensor (Table 1). The other assumption was that the angle between the sensor and object will be fixed. It was also assumed that the temperature of operation will be in the range of 0–50 °C and the sensor will not operate in a very dusty or hazy environment.

**Figure 9:**
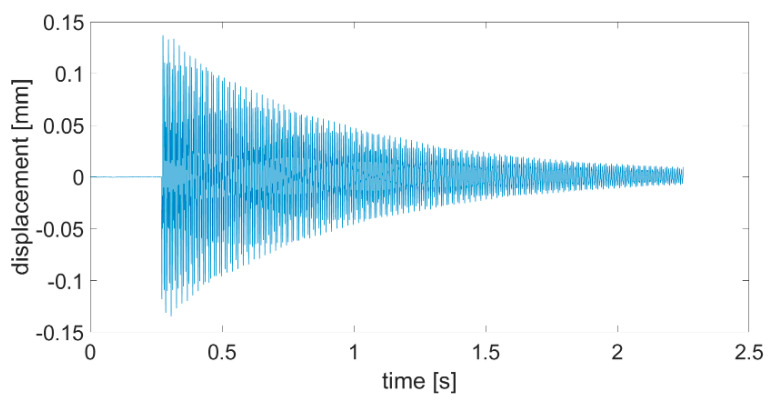
Time course of the P2 plate displacement after impact with a modal hammer.

An example of the plate vibration displacement recorded with a laser sensor after the impact with a modal hammer is shown in Figure 9. The obtained time waveforms were then analyzed in the Matlab software. The main purpose of the analyses was to determine the course of natural vibration frequency changes during the measurement signal damping. Significant changes in dynamic characteristics may indicate non-linearity—damage of the tested object.

## 3. Results and Discussion

**Figure 10:**
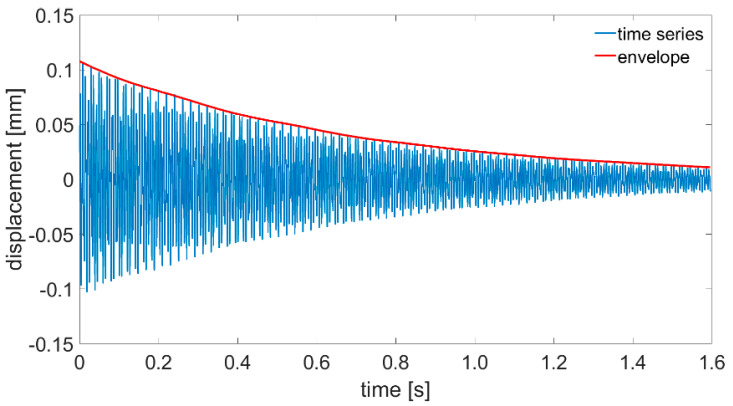
Time course of the P1 plate displacements with the envelope of positive values determined.

The analyses of the recorded vibration signals were carried out in three stages. In the first stage, the analyses of plate displacement signals recorded using a laser vibrometer were performed. The signals were firstly analyzed in a time domain. A new method of damping decrement description was proposed. First of all, as a starting point, a begging of a positive slope greater than the background noise was set for all time domain signals recorded with the laser sensor. Then, the envelopes for positive values were created (Figure 10). 

**Figure 11:**
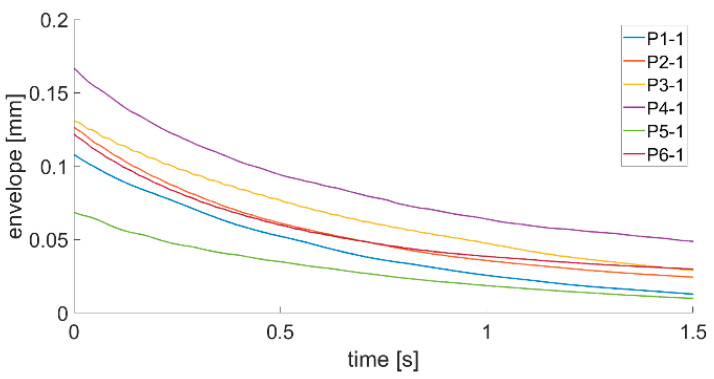
Envelope course of the P1–P6 first series of measurements.

**Figure 12:**
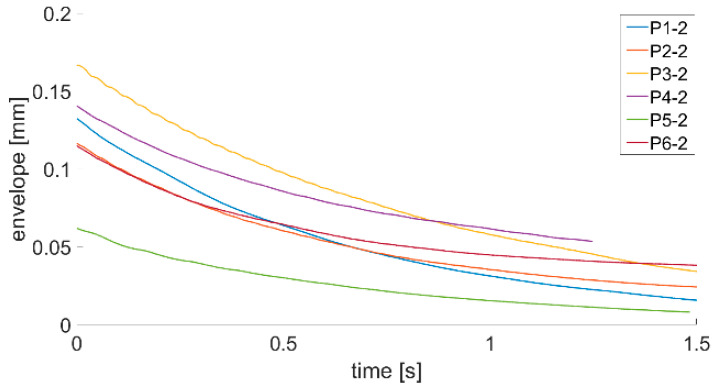
Envelope course of the P1–P6 s series of measurements.

**Figure 13:**
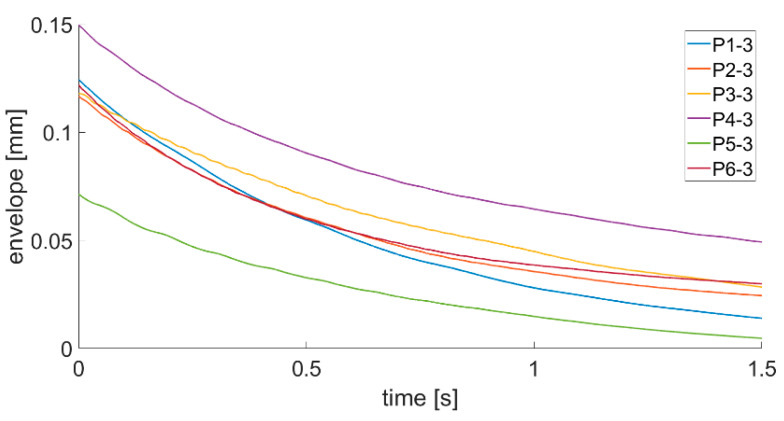
Envelope course of the P1–P6 third series of measurements.

To calculate a damping decrement, a well-known equation was used, but we changed the values of specific amplitudes to values of the envelope at specific time points.

(3) $$\Lambda_{t}=ln\left(\frac{A_{0}}{A_{1}}\right)$$

where $\Lambda_{t}$ is the time logarithmic damping decrement, $A_{0}$ is the value of the displacement time series envelope at the first moment of plate response and $A_{1}$ is the value of the displacement time series envelope after 1 s of the plate’s first response. Such method of damping ratio calculation should be easier to implement in an SHM system installed onboard a ship due to the fact that the system will not have to find values of specific amplitudes. Calculated envelopes for all plates being investigated are presented in Figure 11, Figure 12 and Figure 13.

By analyzing the envelopes’ curves presented in Figure 11, Figure 12 and Figure 13, it can be observed that the time of decay of vibrations resulting from the impulse impact differs for individual plates. Data collected in this step of analysis were used with Equation (3) to calculate the damping decrement. Results are presented in Table 2. 

Analysis of data presented in Figure 11, Figure 12 and Figure 13 and in Table 2 allow stating that the proposed method of damping decrement determination provides results which are different for different technical conditions of welded joints in the analyzed plates. It might be used in an SHM system installed onboard a ship to detect changes in welded joints’ technical condition. If the damping decrement value of a hull in good condition in the place where a laser vibrometer will be installed is known, continuous tracking of its value changes will allow detecting abnormalities in the hull structure.

**Figure 14:**
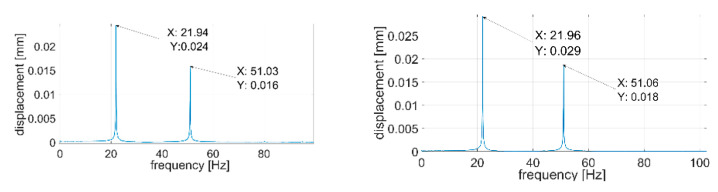

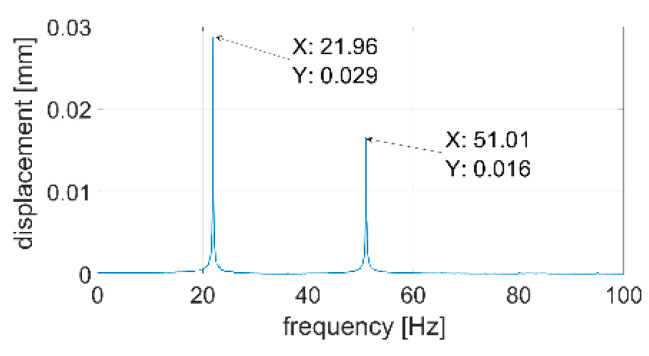
Vibration displacement spectra of P1 plate, signals recorded by a laser vibrometer.

**Figure 15:**
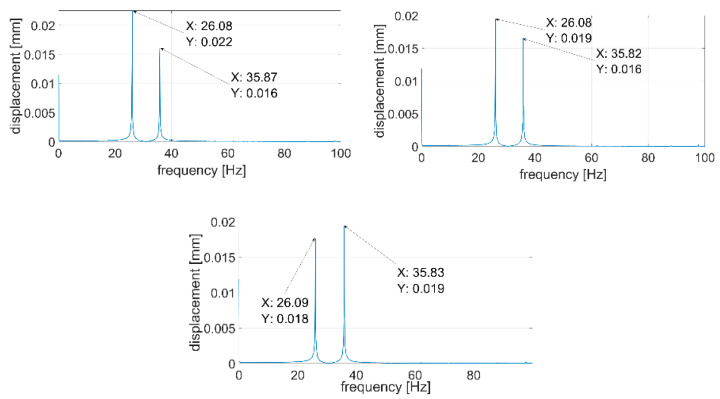
Vibration displacement spectra of P2 plate, signals recorded by a laser vibrometer.

**Figure 16:**
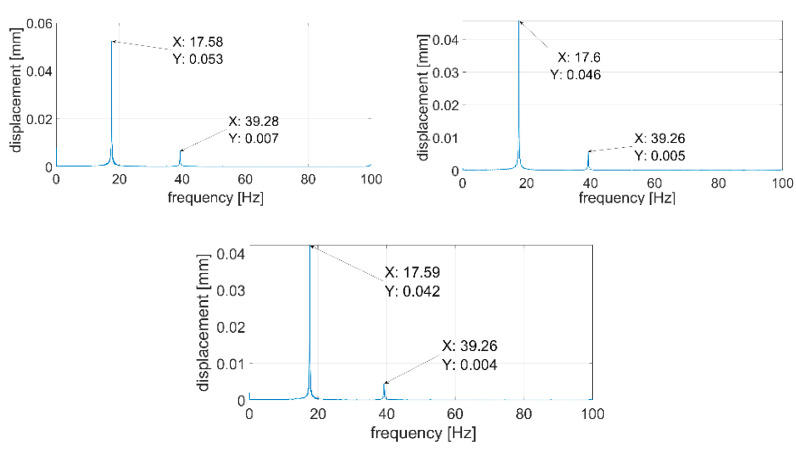
Vibration displacement spectra of P3 plate, signals recorded by a laser vibrometer.

**Figure 17:**
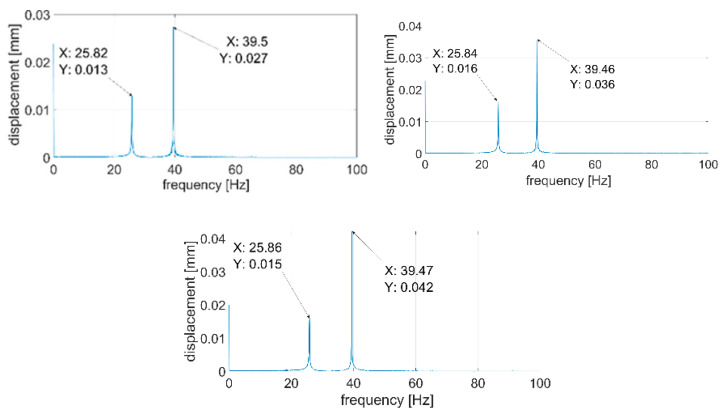
Vibration displacement spectra of P4 plate, signals recorded by a laser vibrometer.

**Figure 18:**
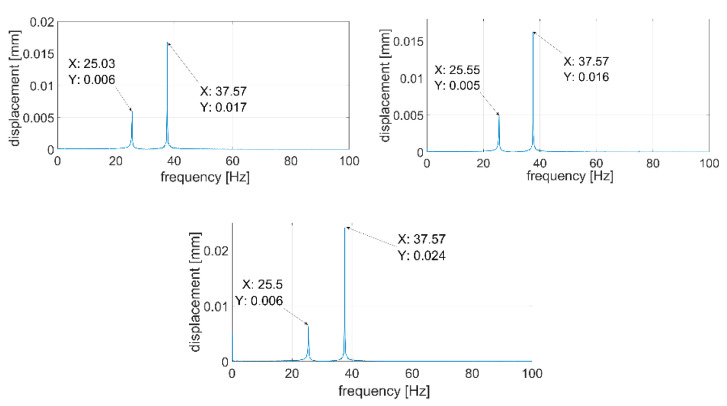
Vibration displacement spectra of P5 plate, signals recorded by a laser vibrometer.

**Figure 19:**
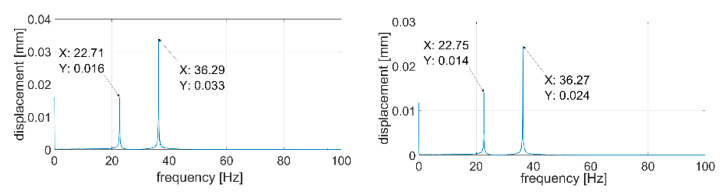

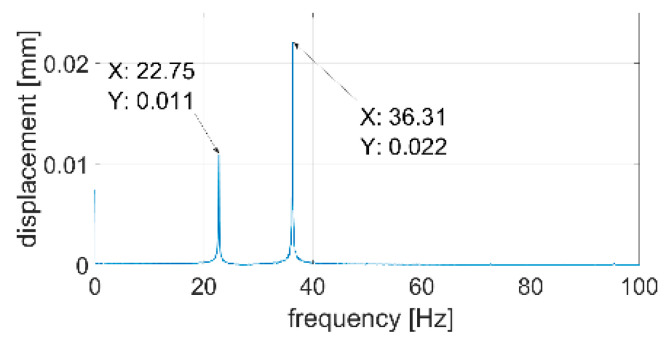
Vibration displacement spectra of P6 plate, signals recorded by laser vibrometer.

During the next step, signals were analyzed with the use of the FFT algorithm. The recorded signals turned out to be of such good quality that it was unnecessary to subject them to filtration or other forms of post-processing. All the recorded waveforms were analyzed (10 for each of the plates—3 of them are presented in the paper), obtaining very high repeatability in the frequency domain in relation to individual plates. In the further part of the work, three waveforms for each of the plates are presented—Figure 14, Figure 15, Figure 16, Figure 17, Figure 18 and Figure 19.

Figure 14, Figure 15, Figure 16, Figure 17, Figure 18 and Figure 19 show the values of vibration amplitudes of P1–P6 plates obtained during the measurements with a laser vibrometer. It was observed that the frequency values of the analyzed forms of the plate’s own vibrations vary depending on the technical condition of the plate. Average frequency values from the three measurements are collected in Table 3.

The results obtained by means of the simulation are characterized by an error of less than 2% (P1 plate) compared to the results obtained when measuring a plate without a welded joint. During the numerical simulation, attempts were also made to model the weld with defects correctly. Local stiffness changes caused by defects and the weld itself are such a complex issue that the analysis becomes extremely complex, which disqualifies this method as a validation support when measuring repeatable weldments. In case of implementation of this method, it will be necessary to thoroughly examine the form of natural vibrations of several elements which are certain of the correctness of welds. On this basis, it is possible to determine limit values, in which the frequencies of the individual forms must be included. The diagnostic method will base on comparing the spectra of the tested object with historical spectra of the same object (in the case of an SHM system installed onboard a ship) or spectra of other objects produced as a series (in the case of a damage detection system on the production line). The analysis of the presented data (Table 3, Figure 14, Figure 15, Figure 16, Figure 17, Figure 18 and Figure 19) shows unequivocally that the occurrence of a properly executed weld (P3 plate) changes the values of the frequency of occurrence of the first two forms of natural vibration compared with a plate without a weld. This is particularly noticeable in the case of the first form of natural vibration, where the P3 plate achieves values at least 30% lower than those of other plates. The comparison of the values obtained in the case of P2, P3 and P5 plates, whose welds are characterized by the same type of defect (no re-melting), with the remaining ones also allows concluding that the type of defects occurring in the weld has such a significant influence on changes in the local stiffness of the material that it is observable in changes in the frequency of the first form of natural vibration (especially the first form).

A very important issue is also the high repeatability of the obtained frequency values of both forms of natural vibrations for all the tested plates. The lack of repeatability of the accompanying amplitudes results from the adopted measurement methodology, where the extortion with a modal hammer was carried out manually, with different extortion forces. In the case of automation of the process, it will probably also be possible to obtain the repeatability of values of amplitudes of vibration displacements.

**Figure 20:**
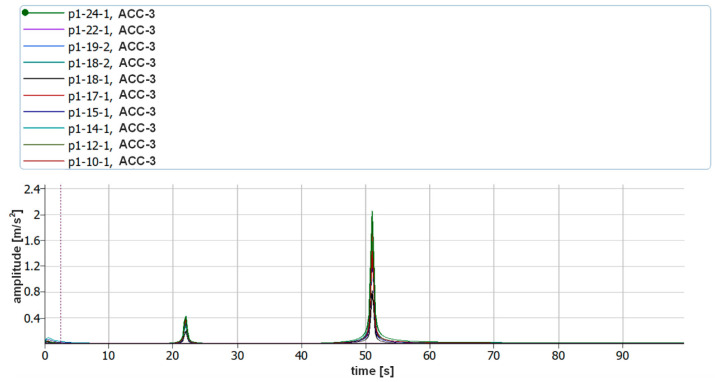
Vibration acceleration spectra corresponding to 10 excitations for the first two forms of natural vibration of P1 plate (ACC3).

**Figure 21:**
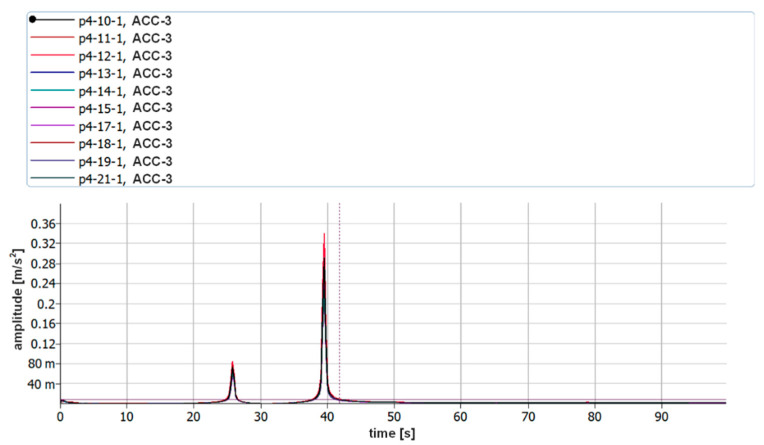
Vibration acceleration spectra corresponding to 10 excitations for the first two forms of natural vibration of P4 plate (ACC3).

The assumption of the measurements was to use two different measuring methods at the same time, which was to enable verification of the correctness of the measurements carried out using a laser vibrometer. Piezoelectric accelerometers were attached to the object tested using bases equipped with neodymium magnets. In order to eliminate possible errors that may occur during the analysis of vibration displacement recordings in the Matlab environment, the analysis of vibration acceleration parameters was additionally carried out with the use of the dedicated Pulse Reflex software from B&K. During these verification analyses, the resolution in the frequency domain was limited to 0.25 Hz. Figure 20 and Figure 21 show the vibration acceleration spectra of the first two forms of natural vibration of P1 and P4 plates. The full frequency repeatability of their occurrence is visible.

**Figure 22:**
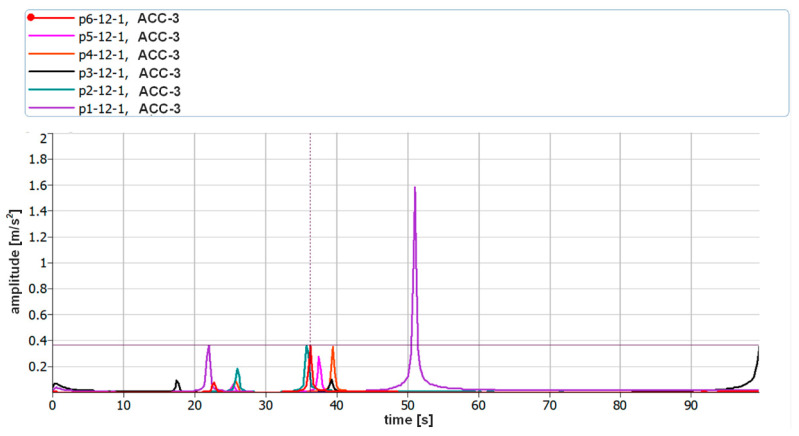
Comparison of the vibration acceleration spectra of all the tested plates (ACC3).

Figure 22 shows the vibration acceleration spectra of all the tested plates with a limit of 100 Hz. The values of the frequencies of the first two forms of natural vibration are collected in Table 4.

The obtained frequency values of the vibration acceleration amplitudes recorded with the use of accelerometers confirm the correctness of the measurements of vibration displacements recorded with a laser vibrometer. Moreover, the commercial software used during the verification indicates that no errors were made both during the simulation and in the first stage of the research. The obtained frequency values are burdened with a small, negligible error.

## 4. Conclusions

In the literature available to the authors, no descriptions of the use of laser vibrometry in diagnostics of welded joints onboard ships were found. The combination of measurements of displacement of welded elements stimulated by a force pulse with the analysis of frequency changes of the first two forms of natural vibration offers promising results. Further, the new proposed method of a damping decrement calculation provides very promising results. After additional verification tests on other objects, methods can be implemented as a tool for quick weld diagnostics, also during the operation of the object. This applies especially to small welded elements. The most important conclusions might be considered as those shown below:

1. Both methods based on damping decrement calculation or displacement spectra analysis require knowledge of parameters describing the object with a known, good technical condition. Therefore, these are qualitative diagnostics methods that enable the detection of damage without indicating its specific cause. According to this, we mean that a new ship (or its element) should be tested with the proposed method before exploitation in order to obtain reference spectra of critical hull points.
2. It should be noted that the methods have the character of non-contact measurements and therefore they can also be used on production lines, where the entire diagnostic process can be automated.
3. During the measurements, a modal hammer was used as the source of the impact signal, so it must be concluded that similar good results will be obtained in the case of impulse extortions implemented in other ways (e.g., stochastic environmental extortion).
4. Undoubtedly, before starting to test a series of products, it will be necessary to know the form of the object’s own vibrations. For this purpose, it will be necessary to test several specimens of a known technical condition.
5. It will be advisable here to use two independent methods of determining dynamic parameters (one of them may be a numerical analysis based on the finite element method (FEM)). Obtaining the frequency value of the first two forms of the object’s own vibrations with welds without defects will allow for testing the remaining ones using only a laser displacement sensor.
6. In the low-frequency range, direct measurement of displacement amplitudes (laser sensors) shows an advantage over the measurement of acceleration amplitudes (accelerometers). This is mainly due to the fact that there is no need to double integrate the acceleration waveforms.

## Figures and Tables

**Table 1:** ILD2300-10 sensor data.

Type ILD 2300-10	
Measurement range [mm]	10 (start of measurement 30, end of measurement 40)
Resolution at 20 kHz [µm]	0.15
Light source	1 mW laser diode with a light output wavelength of 670 nm
Power supply [V]	24
Marker size at the beginning of the measuring range [µm]	75 × 85
Marker size at the end of the measuring range [µm]	110 × 160

**Table 2:** Damping decrement values calculated with Equation (3).

Plate Number	Envelope Value at t = 0 s	Envelope Value at t = 1 s	Damping Decrement
P1	0.108	0.026	1.434
	0.133	0.031	1.442
	0.125	0.028	1.442
P2	0.127	0.036	1.267
	0.117	0.035	1.191
	0.119	0.032	1.313
P3	0.131	0.047	1.013
	0.167	0.058	1.058
	0.118	0.045	0.966
P4	0.167	0.064	0.958
	0.141	0.062	0.825
	0.150	0.065	0.845
P5	0.069	0.019	1.303
	0.062	0.015	1.389
	0.067	0.018	1.314
P6	0.122	0.039	1.150
	0.115	0.045	0.942
	0.117	0.043	1.003

**Table 3:** Frequency of the first and second forms of natural vibration obtained during the simulation and measurement of displacements.

Mode	Simulation	P1 Plate	P2 Plate	P3 Plate	P4 Plate	P5 Plate	P6 Plate
	[Hz]						
I	22.2	21.95	26.08	17.59	25.84	25.36	22.74
II	50.45	51.03	35.84	39.27	39.48	37.57	36.29

**Table 4:** Frequency of the first and second form of natural vibration obtained during the analysis of acceleration parameters of the tested plates.

Mode	P1	P2	P3	P4	P5	P6
	[Hz]					
I	22.00	26.00	17.50	25.75	25.50	22.75
II	51.00	35.75	39.25	39.25	37.50	36.25

## Data Availability

Data sharing not applicable.
